# Intensive home care packages for people with dementia: a realist evaluation protocol

**DOI:** 10.1186/s12913-018-3630-8

**Published:** 2018-11-01

**Authors:** Fiona Keogh, Maria Pierce, Karen Neylon, Padraic Fleming

**Affiliations:** 1 Centre for Economic and Social Research on Dementia (CESRD), ILAS Centre, National University of Ireland Galway, Newcastle Road, Galway, Ireland; 2 Genio, 19-21 Westland Square, Dublin 2, Ireland; 3 Health Intelligence Unit, HSE Dr Steevens Hospital, Dublin 8, Ireland

**Keywords:** Dementia, Home care, Person-centred care, Realist evaluation, Study protocol

## Abstract

**Background:**

Dementia presents a significant challenge to health systems and to the person and family affected. Home care is increasingly seen as a key service in addressing this challenge in a person-centred and cost-effective way. Intensive Home Care Packages (IHCPs) were introduced in Ireland to provide personalised and high levels of support for people with dementia to remain at home or be discharged home from hospital, and to build on the work of the HSE & Genio Dementia Programme. This realist evaluation is concerned with real world questions of feasibility and effectiveness; specifically understanding in what ways IHCPs work, how optimum outcomes are achieved, for whom and in what contexts do IHCPs work best.

**Methods:**

A mixed-method, multi-stakeholder study was designed within a realist evaluation conceptual framework. The process evaluation includes semi-structured interviews with health service staff at all levels, social network analysis and secondary database analysis; the outcomes evaluation includes quantitative measures and qualitative data collected through in-depth interviews with people with dementia and family carers; and the cost evaluation includes analysis of data from the Resource Utilisation in Dementia (RUD). The four stage cycle of realist evaluation is adopted, with iterative rounds of theory formulation, data collection and theory testing throughout.

**Discussion:**

This realist evaluation of a complex intervention involves a variety of data and perspectives in order to provide confidence in moving from hypothetical constructs about how IHCPs might work to explanations of potential or observable causal mechanisms. In spite of being a key form of service delivery in most healthcare systems, the ways in which home care works to produce the desired outcomes seems to be poorly understood. While there is much descriptive and comparative work, there is a lack of understanding regarding which patient groups might benefit most from home care, or the influence of different service or cultural contexts on outcomes from home care. As well as addressing the core research objectives, this study aims to make a contribution to the underlying theory of home care in ways that can progress our understanding of how outcomes are produced for home care recipients.

## Background

Dementia is an age-related condition. The challenge posed by dementia, which is the confluence of increasing life expectancy, population growth and the lack of curative treatments, has been well documented [42, 54]. The overall societal cost of dementia is high, estimated at US$818 billion globally [42] and at €1.69 billion in Ireland [8]. Although the personal impact of dementia has been documented for both the individual and the carer [25], there is less attention on the combination of services and supports needed by the person and family throughout the dementia journey or on their views of what works for them [11].

It is the preferred wish of most people with dementia to continue living in their own homes for as long as possible. In December 2014, the Irish government published its first National Dementia Strategy (NDS), which supports this preference [13]. It stated that: “People with dementia should be facilitated to remain living in their own homes and to maintain existing roles and relationships for as long as possible …” (p. 24). The provision of integrated home care services is a priority action of the Strategy, which is underpinned by the dual principles of personhood and citizenship.

The main services underpinning the policy aim of supporting people with dementia to remain at home are home help services and the Home Care Package (HCP) scheme. Provision of hours under these schemes is rationed, with an emphasis on task-oriented care [12], illustrated by the half-hour or hourly slots typically allocated for home care workers’ time with clients. People with dementia are high users of these services [33], but reliable data on the receipt of home care by people with dementia are not available nationally [6]. The community care system in Ireland, which includes home care services, has been described as underdeveloped and fragmented, with a small range of services and inconsistent availability [6]. While the current community care system offers limited scope for providing home care that is person-centred, in recent years new service models aimed at providing more person-centred community-based care have been developed and tested in the HSE-Genio Dementia Programme. This programme is a collaboration between the Health Service Executive (HSE), Ireland’s national health service, and Genio, an NGO supporting health service innovation [18]. Although policy over 50 years has advocated community-based services to enable people to age well at home, resource allocation is skewed, with 75% of funding for older people being spent on residential care [34]. The bulk of care for people with dementia living at home is provided by family members. The largest proportion of cost falls on family or informal carers (48%), with 43% attributed to residential care costs [8]. This cost breakdown largely aligns with other countries in Europe [56].

Dementia is common among older people admitted to acute hospitals; about 29% of older people admitted to public hospitals in Ireland have dementia [52]. People with dementia typically have longer length of stay in acute hospitals [9, 52] and their outcomes are generally poorer than people without dementia [46] A comprehensive, integrated, well-resourced system of community care services, including home care, is required to support people with dementia to remain living at home for as long as possible and to facilitate timely discharge home from acute hospital admission [54].

### Evidence on home care services

The evidence on community based services supporting people with dementia living at home is limited and systematic reviews point to many gaps in the evidence base [11]. The best outcomes for people with dementia are associated with services that are timely, responsive, flexible and tailored to individual need [11]. Specialist multiagency home support has been shown to provide flexible, responsive care that involves people with dementia in decision-making and enables relationship building, thereby promoting personhood [44]. Systematic reviews have noted the heterogeneous nature of social care interventions, populations and methodologies and the challenging nature of conducting effectiveness research in this area [2].

Optimum dementia care is complex, necessitating a multitude of services and supports from a range of providers, in a variety of settings, to meet the medical, personal care, social and psychological needs of people with dementia, in addition to providing responsive support to family carers. The health and social care system itself is complex and fits the definition of a complex adaptive system “…a collection of individual agents with freedom to act in ways that are not always totally predictable, and whose actions are interconnected so that one agent’s actions changes the context for other agents” [41](p.625).

### The initiative: Intensive Home Care Packages (IHCPs) for people with dementia

The Health Service Executive (HSE) introduced Intensive Home Care Packages (IHCPs) for people with dementia at the end of 2014 as one element of a range of initiatives to address pressure on acute hospital beds. The IHCP Initiative is not typical of well-defined interventions with distinct boundaries, but is more akin to interventions that [48] characterise as highly complex, large-scale and ‘messy’ and which ‘require imaginative approaches to evaluation that go beyond assessing progress beyond predefined goals and milestones’ ([19]: 391). Rather than being a distinct addition of a ‘new’ intervention to publicly-funded home care services, the IHCP initiative could be better described as a change effort in which a set of ideas are being tried to bring about health and social service transformation in a highly complex health care system. Accordingly, the initiative had multiple aims. Chief among them was to facilitate timely discharge home from acute hospitals, for people who require “very significant interventions to an extent not previously provided as part of the HCP Scheme or current community services” [22]. It was also intended that the scheme would be available to people living in the community to prevent unnecessary hospital admission. The Scheme was primarily aimed at older people but it was also used for some people under 65 years of age (for example, early onset dementia or other neuro-degenerative disorders). This initiative is closely aligned with the NDS goal of supporting people with dementia to remain living well at home and a funding package to deliver up to 500 dementia-IHCPs over a three-year period was made available as part of the implementation of the NDS.

At the individual level, the intention was that the content and delivery of IHCPs would not just provide more support, but would provide a wider range of supports and would be qualitatively different from usual home supports, building on the work of the Genio & HSE Dementia Programme, which developed and tested personalised community based supports for people with dementia [18]. The IHCPs aimed to be flexible in their design and delivery, and tailored to the individual person’s assessed physical, psychological and social needs. The range of supports and services to be provided could include, for example; home care hours to provide personal care, supervision and maintenance of personhood and life roles; nursing and/or allied therapy interventions; aids and appliances; respite care including in-home respite; and overnight care. There was also a strong emphasis on supporting family carers. The IHCPs could be provided short, medium or long-term, depending on assessed need and regular review. The different elements of the package were delivered either by trained health and social care professionals or by home care workers, the latter employed either directly by the HSE or by an approved private home care provider, who have basic, generic training and may (or may not) have training in dementia care. The level of funding available for IHCPs was substantial compared to what was typically available at that time; between €850 to €1,500 per person per week. The lower funding limit of €850 per week was provided to distinguish these packages from existing HCPs, which had an upper funding limit of €525 per week, highlighting the difference in quantum and content from current provision. At the outset, IHCPs were made available to people in nine identified acute hospitals and their catchment areas. In addition to the targeted funding, a Standard Operating Procedure (SOP), developed by Senior Managers in the HSE, was made available to HSE staff tasked with implementing the IHCP scheme.

As the IHCP initiative was emergent and dynamic, after the first year of operation several changes took place, specifically in relation to eligibility criteria, geographical areas targeted and funding thresholds. The eligibility criteria were clarified to include the support of people with dementia to remain in the community and prevent frequent acute hospital attendances/admission to residential care, as well as facilitating timely discharge from acute inpatient care. The target areas were expanded to include more hospitals. The lower funding threshold, which had created a gap between the upper limit of the HCP and the lower limit of the IHCP, was revised down to €700 per week and later to €500 per week to address the anomaly created by the gap.

### Evaluation rationale and aims

While the initiative was designed to address a specific need for service, there was a broader intention to test the feasibility of this type of support and evaluate its effectiveness at supporting people with dementia who had a high level of need to remain at home. The high level evaluation questions focused on; practical effectiveness or feasibility - whether the initiative works in everyday practice for the target group; user satisfaction, impact on quality of life (QOL) of the person and family carer, and cost-effectiveness. Thus, outputs required for policymakers and service funders were those that would describe process, outcomes and costs. The goals and objectives of the evaluation of IHCPs for people with dementia were to:

1. Examine the existing arrangements that have been developed nationally for the delivery of IHCPs for people with dementia around the country, from which the key components and characteristics of packages will be identified and their association with specific outcomes for people with dementia and their family carers (for example; time at home on IHCP, QoL, carer burden and satisfaction).
2. Contribute to an understanding of ‘what works, for whom, under what circumstances’ with respect to IHCPs for people with dementia, including from the perspective of people with dementia and their family members, identifying contexts in which the IHCPs achieve (or fail to achieve, as the case may be) the anticipated benefits/outcomes and the mechanisms contributing to observed outcomes.
3. Establish the costs of IHCPs for people with dementia from both a funders (HSE) and a societal perspective and determine the cost-effectiveness of IHCPs vis-à-vis acute hospital care and long-stay residential care.

The perspectives of multiple stakeholders are fundamental to generating a complete understanding of the initiative. A particular emphasis is placed on including people with dementia as much as possible and the research team consulted with the Irish Dementia Working Group (IDWG) on the research questions and methods for this study.

## Design and methods

The IHCP initiative commenced in 2014 approximately 1 year before the evaluation was designed. A data set on all IHCPs has been collected from the outset and other data collection commenced in 2016. At the time of writing data collection is still underway. Due to the complex nature of the initiative and the system within which it is being implemented, further compounded by (i) the lack of accessible comparison groups to conduct a controlled trial; (ii) the need for evidence that addresses the variability in the population of interest (people with dementia and family carers); (iii) the variability in the delivery IHCPs; and (iv) changes to the initiative in response to the initial roll out; a realist evaluation design was deemed most appropriate to address the objectives of the evaluation. A realist approach was also deemed the most likely to yield relevant outputs to inform decision-making by policymakers regarding the future development of IHCPs and implementation of personalised home care more generally.

Realist evaluation is increasingly used in the assessment of complex interventions [58]. A realist approach [36–38] aims to understand what works, for whom, in what circumstances and to what extent. It operates at the ‘middle range’ “using concepts that describe interventions at a level between big policy ideas and the day-to-day realities of implementation” ([39], p.18), hence it is particularly apposite for an evaluation of IHCPs. The reporting standards for realist evaluation informed the study design [58].

A comprehensive, multi-stakeholder evaluation of the process, costs and outcomes of IHCPs was designed. Typically, a realist evaluation is structured according to realist methods described by Pawson and Tilley [38] and as applied by others (for example, [30, 43] in their realist evaluation protocols; *Stage 1* - develop an understanding of the intervention and develop initial programme theory; *Stage 2* - collect data to test these theories and address the core research questions; *Stage 3* - analyse data to interrogate the theories; and *Stage 4* - interpret findings to explain, revise and refine the initial programme theories and the intervention itself and to develop a refined middle range theory (MRT).

**Fig. 1:**
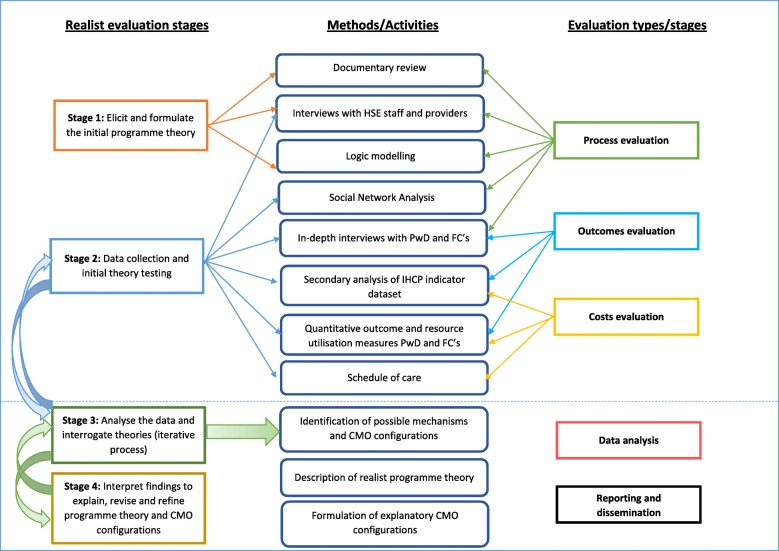
Interrelated components of the methodology

The evaluation is best described as a nested design, with process, outcome and cost evaluations sitting within a realist framework. Figure 1 shows the different components of the study each of which is described in detail below and the ways in which they relate to the different stages of the realist evaluation.

### Stages 1 & 2: Develop an understanding of the intervention, develop initial programme theory and data collection

There is considerable overlap between the methods used to collect the data required for stage 1, understanding the intervention and theory development, and stage 2, collecting data to test the theories. Therefore the methods for both are described here. The methods are grouped into process evaluation, theory development, outcome evaluation and cost evaluation, although there is overlap in how the data is used as illustrated by Fig. 1.

#### Process evaluation

A process evaluation was designed, informed by the Medical Research Council (MRC) guidance on *Process Evaluation of Complex Interventions* [31]. Data from the process evaluation are being used for several purposes: to contribute to a detailed description of the programme theory and underlying assumptions; to capture how the IHCPs are delivered in practice; to provide a description of potential mechanisms; and to provide a detailed consideration of contextual factors shaping the development and operation of the programme. The process evaluation adopts a mixed-methods approach, with five key elements, described in detail here:

(i) *Documentary review*: Documents to be reviewed include the National Dementia Strategy Implementation Plan (NDSIP), the Standard Operating Procedure (SOP), National Guidelines for Home Care Packages, and any other documents relevant to the development and delivery of IHCPs and evaluations of HCPs for older people [10, 32, 35, 53]. The documentary review is being conducted to assist in the identification of key aspects of the scheme and the process issues that would be worth exploring further [31] and to identify potential mechanisms and descriptions of contexts to begin the process of identifying Context-Mechanism-Outcome Configurations (CMOCs) to inform data collection at later stages.
(ii) *Interviews with key HSE staff and external service providers*: These interviews are qualitative semi-structured interviews guided by a topic schedule, designed to obtain information to describe and formulate the programme theory, particularly contexts and mechanisms. Care was taken to remain open to other relevant information. As recommended by [28] a variety of key HSE staff involved in the management and delivery of IHCPs are being interviewed, including those involved at operational level, in the application, assessment, care planning and approval processes, followed by those involved in delivering and reviewing IHCPs. Interviews with a selection of external home care providers delivering supports to people with dementia under the IHCP will also be undertaken. Certain key staff will be interviewed a second time to test the theories that are emerging from the data analysis.
(iii) *Social Network Analysis* (SNA): SNA is used to investigate relationships between various actors, the structural patterns of relationships that exist and the impact that these have on service delivery [16]. Drawing on the interviews with HSE staff and external service providers, visual representations of the social networks of the various actors involved in providing IHCPs will be undertaken. Social networks are useful as a quick means of representing organisational structures. Network analysis can contribute to a greater understanding of communication and collaboration between service providers, and can draw attention to the types of relationships that generate communication and learning, which may sometimes be unexpected and less hierarchical than envisaged in the design stages. This is important data in elucidating context and the ways in which mechanisms may be operating. For example, is the network of workers involved in an IHCP for a hospital discharge more complex than the network involved in a community IHCP and how might this influence the content and effectiveness of the IHCP?
(iv) *Secondary analysis of IHCP indicators dataset*: As part of the IHCP initiative an indicator framework was developed to assist in measuring ongoing costs and progress towards individual outcomes. This framework has seven outcome domains; individualised, effective, efficient, accessible, safe, fit for purpose and sustainable. Each domain has indicators to reflect the perspective of the person supported by the IHCPs, the carer and the wider service provision system (i.e. HSE), as relevant. The indicators are being used by the HSE to measure the quality of IHCPs. The coded and anonymised indicator dataset has been made available to the evaluation team for secondary data analysis. In addition to the indicator data, data is also collected on age, sex, marital status, main informal carer, living arrangements, dependency level (measured using the Barthel Index), the geographical location (HSE region and county), referral source (i.e. from community or hospital), specific components and duration of the IHCP, reasons for cessation, resource use, approved and weekly costs. This data will be important in describing the content of the IHCPs themselves and in the understanding of context and outcomes.

##### Initial programme theory development

**Fig. 2:**
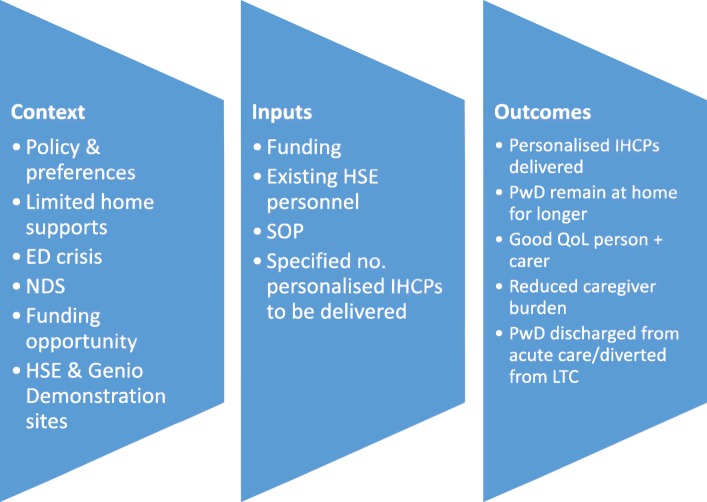
Initial programme theory for IHCP initiative

*Logic Modelling:* As recommended by the MRC guidance [31], logic modelling is being used to develop a thorough understanding and description of the initiative and to develop programme theory. It is a useful method for explicating the inputs and expected outputs of a programme and for identifying underlying assumptions [24]. Data from the process evaluation such as the documentary review and the qualitative interviews with HSE staff will be used to develop the initial or formal programme theory, i.e.; a description of context, inputs and expected outcomes of the initiative (see Fig. 2). In line with realist evaluation, this initial programme theory will be refined and understanding of context and potential mechanisms will be developed as interviews with key HSE staff are completed and as theory building progresses. An example of how the data from the process evaluation will contribute to this theory development is in a consideration of home care hours in the IHCP. In a process evaluation, the distribution of these hours is important in terms of how and why they might be different between different groups. This information is valuable in itself and tells us about the process of care. Using a realist approach, we can also focus on how the provision of the hours for example, affects the families’ willingness or ability to care. Here we are trying to get at the mechanism that might be operating in reducing carer burden (or not) in the context of high or low levels of hours of care.

Following the development of the initial programme theory (Fig. 2), the next stage is to develop this into a realist programme theory and to test it using the evidence collected during the evaluation. One strategy we propose to adopt is to use concepts from existing formal theories as a framework for building the realist programme theory. Family carer burden offers an example. Reducing caregiver burden is an intended outcome of the IHCP Initiative. While we can measure potential reductions in burden, this study will provide an opportunity to examine how the initiative might lead to this outcome, For example, whether it will work to reduce carer burden for some family carers but not others (e.g. spousal carers and adult children). The caregiver stress process framework [40] will be used for building and testing this aspect of the realist program theory, and making explicit for which family carers the initiative works, in which circumstances and how.

### Outcomes evaluation

(i). *In-depth interviews with people with dementia and family carers:* It is expected that IHCPs will be provided to up to 300 people with dementia in the course of the three year initiative. The aim is to conduct in-depth interviews with a sample of between 40 and 60 dyads, i.e. people with dementia supported by an IHCP and their family carer. Participants are invited from all sites as contexts are expected to be different across sites. Where the package has been in place for some time, participants are invited for one interview (retrospective group). For newly implemented packages, participation will involve two meetings with the researchers; a baseline meeting before or shortly after the IHCP commences and a follow-up meeting about eight weeks after this first interview (prospective group). It is hypothesised the experience of an IHCP for the person and carer will change over time. Using this sampling approach, a variety of contexts and potential mechanisms can be captured.

All potential participants receive an invitation to participate and information sheet with detailed information about the study, provided in lay language. Interviews are arranged at a time and location that is convenient to respondents who consent. Reassurances regarding confidentiality are provided. Participants are asked to sign a consent form before the interview commences. Further details on ethical issues and particularly the consent process are provided below. Both quantitative and qualitative data is collected at these interviews:

(ii) *Quantitative data collection:* A structured questionnaire has been developed to collect quantitative information, which will be collected by researchers during interviews with a sample of between 40 and 60 people with dementia in receipt of an IHCP and a family member. The questionnaire comprises two parts. The first part relates to the person with dementia and collects socio-demographic information as well as information on quality of life, health (i.e. comorbidity, falls, dementia sub-type and years diagnosed, dementia severity, activities of daily living) and user satisfaction. Logsdon’s QOL-AD tool was used to derive a measure of the quality of life of the person with dementia [26, 27]. The QOL-AD is a brief 13-item tool, designed specifically to obtain a measure of the person with dementia’s quality of life (QOL) from the perspective of both the person with dementia and the family caregiver (Logsdon & Albert, 1999). Comorbidity is measured using a list of 13 common conditions, based on the Comorbidity Questionnaire [47]. As recommended by ICHOM (2016), dementia severity is assessed using the Dementia Severity Rating Scale, an informant-based, multiple-choice questionnaire that assesses severity from the mildest to the most severe stages in 12 major functional and cognitive domains affected in dementia [7]. The Bristol Activities of Daily Living Scale, developed specifically for use with people with dementia, is used to to reveal the everyday ability of people who have memory difficulties. It is a carer rated, multiple-choice instrument consisting of 20 daily living activities [4].

The second part of the questionnaire relates to the family carer and collects socio-demographic information as well as information on the family carer’s health, health-related QOL, caregiver burden, and satisfaction with IHCP. General health is assessed using a single question rated on a five-point Likert scale, i.e. Excellent, very good, good, fair, poor. Carer-reported health-related QOL is assessed using EQ-5D-3 L published by the EuroQOL group. [3, 20]. The Zarit Burden Interview (ZBI) is used to measure the impact of caregiving on the health, personal and social life, psychological wellbeing and finances of family carers [59–61]. These instruments were administered at the baseline and follow-up meetings. As well as addressing specific evaluation questions, these quantitative outcomes will inform the development of CMO configurations for testing and refinement.

(iii) *Qualitative data collection*: In-depth, semi-structured interviews with people with dementia (if feasible) and their family members are also conducted. The interviews are guided by an interview schedule, piloted with a small number of carers. These qualitative interviews are designed to capture the experience of carers and also to elicit relevant information for theory testing, such as, how some of the programme mechanisms may have influenced their outcomes and what are the contexts in which family carers are providing care. The second interview with some dyads provides an opportunity to explore and test theories and CMOCs as they emerge.
(iv) *Costs and cost-effectiveness evaluation:* Data on costs of IHCPs is available from the indicator dataset and will form part of the secondary analysis. Additional quantitative data on resource utilisation is collected from participants during the in-depth interviews, using the Resource Utilisation for Dementia (RUD) Version 4.0 [55, 57]. The RUD, developed to capture the use of resources by people with dementia, has been widely used in both cost of illness and evaluation studies in several countries including Ireland [8]. The RUD has been adapted with permission to take account of the Irish context and will collect information on the use by people with dementia and their family carers of primary care, community care, out-patient care, and acute care services. Information on medication use by people with dementia and information on caregiving time and employment status of family carers will also be collected. The RUD will be administered as an interview with the family carer in a face-to-face interview. A detailed weekly home care schedule developed by the research team will capture data on the hours and type of care provided by different paid providers and informal carers.

These data will allow us to undertake a costs evaluation of the IHCPs for people with dementia from both a HSE and a societal perspective and to make comparisons across HSE sites delivering IHCPs. Cost-effectiveness of IHCPs for people with dementia vis-à-vis acute hospital care and long-stay residential care will also be determined, thus addressing a central evaluation question. Data from the costs evaluation will also inform the development of CMO configurations.

#### Stage 3: Data analysis

The data collection methods focus on measuring outcomes; eliciting information on context and on the mechanisms that may be operating; how these relate to each other and how they function to produce the observed outcomes. Intrinsic to realist evaluation is an iterative cycle of data collection, theory development and theory testing, leading to further data collection etc., thus the stages 1–3 have not progressed in a linear way, with the second interviews with some HSE staff and family carers being particularly useful in this regard. In the theory development part of this iterative process, a number of steps will be taken to develop Context-Mechanism-Outcome configurations (CMOCs), using outcomes from the initial programme theory to guide this specific process. For each outcome, the steps will be as follows: (i) develop a series of ‘if-then’ statements; (ii) identify contexts and mechanisms within these statements; (iii) list context, resources and reactions separately to identify and formulate mechanisms and then formulate CMOCs; and finally (iv) examine the validity of mechanisms informally in a roundtable discussion by choosing different contexts and discussing whether the specific outcome was ‘switched on’ or ‘switched off’ through the operation of this mechanism. This process will help identify the data to be collected in second interviews in order to test the emerging CMOCs. An example of how this may work is in considering how an IHCP might work in facilitating discharge from hospital. The context includes a hospital that has access to IHCPs, relevant staff are aware of IHCPs and understand the process to put them in place, a family carer is available, the home care provider has capacity etc. The potential mechanism includes both a resource (visible) and a response element (invisible) such that the resource is put in place (home care hours, visits from nurse, information for the family etc.) and the response is a commitment on the part of family and staff to support the IHCP and a belief that it will work. This is one example of how in a specific context the underlying mechanism of commitment and belief can lead to the outcome of a discharge from hospital.

A data analysis plan has been developed. Quantitative data will be analysed using exploratory, inferential and multi-variate methods and this analysis will provide much of the data for the outcome evaluation. Standard regression methods and weighted multiple regression will be used to test relationships of interest. The covariates for this analysis will include: living alone; gender; age at approval; marital status; referral setting; informal caregiving; and Barthel Index dependency. The first phase of qualitative data analysis will be to code the interview data. The different coders on the team will meet to discuss and refine the codes generated. The second phase is data reduction. There are two main approaches to generating codes in realist evaluation. The first is to code the interviews in terms of statements related to contexts (C) (for example norms and values of health services staff, socio-demographic characteristics of clients), mechanisms (M), such as receptiveness of staff to the initiative and outcomes (O) such as length of time at home, drawing on a qualitative thematic analysis process, generating discrete codes for each and using data reduction to determine the associations between contexts, mechanism, and outcomes (Byng et al. [5]. A second approach, (Jackson and Kolla [23], is to generate CMO connections empirically from the data. The focus here is on looking for CMO connections within the interview data, referred to as strings. Dyadic, triadic or more complex strings (CM, MO, CMO strings) are identified in the narratives. Jackson and Kolla [23] used both methods and described the latter as the most efficient and it is this approach which will be adopted.

The cost analysis will use the data from the RUD and the cost of service provision will be calculated by attaching the appropriate unit cost to the relevant averaged resource use across all elements of provision. There is no common, uniform database that covers unit costs in health care in Ireland. Consequently, information on unit costs comes from a variety of Irish data sources. Labour costs will be calculated using consolidated salary scales available from the HSE for public-sector employees, with associated non-pay costs estimated according to the methods outlined by the Health Information and Quality Authority [21]. Duration of visits will be calculated according to the methods outlined in the Regulatory Impact Analysis guidelines issued by the Department of the Taoiseach [21].

### Stage 4: Interpretation

The programme theory which emerged from Stage 1 and the CMOCs emerging from Stage 3 will be assessed and interpreted to determine what has worked, for whom, in what ways and in what contexts. The identification of the characteristics of IHCPs and their association with outcomes for the person and family and the cost-effectiveness analysis will also be considered at this stage. Unexpected or unintended outcomes may provide pointers for further analysis. It is expected that this iterative process, which has run throughout the study, will continue through this stage, with several rounds of theory testing and interpretation. The emphasis will be on making sense of the outcomes in a data driven way.

### Ethical considerations

An important focus of this evaluation was to include the voice of the person with dementia as far as was practicable and for them to be full participants in the study alongside family carers, HSE staff and service providers. The value of including people with dementia has been well described [45]. However, concerns are often expressed regarding the ability of people with dementia to provide informed consent to participate in research [49], thus the ethical issues and the process for approaching and obtaining consent from people with dementia were carefully considered.

The approach adopted for obtaining consent from people with dementia/moderate to severe cognitive impairment for this study is ‘process consent’ [14], which involves five parts: background and preparation, establishing a basis for capacity, initial consent, ongoing consent and monitoring, and support. Consent is obtained at a face-to-face meeting (most likely at the person’s home) and is sought separately from persons with dementia and family carers. Given that people with dementia have different levels of capacity and that this might vary for individuals depending on the day or time of day, the issue of consent and capacity to consent is considered in each instance.

## Discussion

This paper describes a protocol for a realist evaluation of a complex intervention – intensive home care packages for people with dementia (IHCPs), using a multi-method design to understand in what ways IHCPs work, how optimum outcomes are achieved, for whom and in what contexts do IHCPs work best. The design is complex with multiple elements including; process, outcomes and costs evaluations; and multiple stakeholders including; people with dementia, family carers and health care staff from regional and local managers to front line providers. This variety of data and perspective is necessary in a realist evaluation to have confidence in moving from hypothetical constructs to explanations of causal mechanisms.

An evaluation of this nature would typically use a quasi-experimental design, seeking to measure the effectiveness of the IHCP initiative, using different analytic techniques to isolate the effect of different variables on the outcome. However, this evaluation is being conducted in real world, dynamic conditions and is interested in “how variables combine to create outcomes” [15] and specifically, in which conditions and through which configurations of factors, contexts and mechanisms the outcome is achieved [29]. This importance of taking context into account was reinforced by a key conclusion in the large-scale RightTimePlaceCare study in response to the wide variation in findings between countries, that “interventions that aim to reduce caregiver’s burden should take contextual aspects into account” [1]. For these reasons a realist evaluation approach was adopted.

One of the strengths of this approach is that it addresses questions of practical importance for those tasked with implementing service initiatives and for those tasked with commissioning and funding initiatives. Rather than focus on the question ‘does it work’, realist evaluation aims to uncover the mechanisms which contribute to certain outcomes in specific contexts and the findings are therefore potentially more generalisable than trials on highly selected groups in artificial service settings. However, the approach has limitations. The iterative and adaptive nature of the approach makes it challenging to describe the design and methods clearly at the outset. Causality is not demonstrated and there are few ‘neat answers’. A specific limitation of this study is that the sample size for the in-depth study, where the detailed measurement of outcomes takes place, is relatively small. However, the larger data set covers the total population of cases and will potentially allow for some statistical modelling of findings depending on the similarity of the in-depth sample to the larger data set.

As well as being the mode of care preferred by most older people, home care is seen as a potentially cost-effective way of addressing the growing demand for care coupled with the diminishing potential for informal care [17, 50]. However, in seeking to elicit and formulate the MRT or programme theory underlying IHCPs, we were struck by the paucity of research on the ‘theory’ of home care and it seems home care as a phenomenon and concept is poorly understood [51]. There is considerable descriptive ([50, 51] and comparative [17] literature on home care, and some systematic reviews of the effectiveness of home care in comparison to other settings, which suffer from the shortcomings of RCT methods in addressing complex interventions ([2, 11]. However, there is little evidence on the aspects of home care that achieve the desired outcomes, or what outcomes home care can reasonably be expected to achieve, for whom and in what settings or cultural contexts. As well as addressing the core research objectives, this work aims to elucidate what changes are needed to move the home care system to a more responsive, personalised model in which the dignity of the person with dementia and family carer is respected. It is hoped that this work will also make a contribution to the underlying theory of home care in ways that can progress our understanding of how home care can produce more person-centred care and better outcomes for the recipients.

## Abbreviations

ADI: Alzheimer’s Disease International
CMOCs: Context, Mechanism, Outcome configurations
CSAR: Common Summary Assessment Record
ED: Emergency Department
HCP: Home Care Package
HSE: Health Service Executive
ICHOM: International Consortium for Health Outcomes Measurement
IHCPs: Intensive Home Care Package
MAU: Medical Assessment Unit
MRC: Medical Research Council
MRT: Middle range theory
NA: Network Analysis
NDS: National Dementia Strategy
NDSIP: National Dementia Strategy Implementation Programme
NESF: National Economic and Social Forum
PwD: Person with Dementia
QOL: Quality of Life
QOL-AD: Quality of Life in Alzheimer’s Disease
RUD: Resource Utilization for Dementia
SNA: Social Network Analysis
SOP: Standard Operating Procedure
UK: United Kingdom
WHO: World Health Organisation
